# Absorption Study of Mozuku Fucoidan in Japanese Volunteers

**DOI:** 10.3390/md16080254

**Published:** 2018-07-30

**Authors:** Kizuku Kadena, Makoto Tomori, Masahiko Iha, Takeaki Nagamine

**Affiliations:** 1 South Product Co., LTD., Uruma, Okinawa 904-2234, Japan; m-tomori@south-p.co.jp (M.T.); miha.south@nifty.com (M.I.); 2 Graduate School of Health Science, Gunma University, Honorary Professor of Gunma University, Maebashi, Gunma 371-8511, Japan; mine@gunma-u.ac.jp

**Keywords:** fucoidan absorption, urinary fucoidan value, inhabitants of Okinawa prefecture, mozuku consumption

## Abstract

We performed an oral administration study of fucoidan in 396 Japanese volunteers and investigated significant factors concerning the absorption of fucoidan. Urine samples were collected at 0, 3, 6, and 9 h after ingestion of 3 g of fucoidan. Fucoidan was detected in urine after ingestion in 385 out of 396 subjects. The maximum value (mean ± standard deviation (SD)) of urinary fucoidan was 332.3 ± 357.6 μg/gCr in subjects living in Okinawa prefecture, compared with 240.1 ± 302.4 μg/gCr in subjects living outside Okinawa. Compared with the estimated urinary excretion of fucoidan by place of residence, those of subjects living in Okinawa prefecture were significantly higher than those living outside Okinawa prefecture (*p* < 0.01). In addition, subjects living in Okinawa prefecture consumed significantly greater amounts of mozuku compared with those living outside Okinawa prefecture (*p* < 0.01). Multiple regression analysis showed that having Okinawa prefecture as a place of residence was a significant factor (*p* < 0.01) contributing to the estimated urinary excretion of fucoidan. Because the habit of eating mozuku was significantly higher (*p* < 0.01) in subjects living in Okinawa prefecture than in those living outside Okinawa prefecture, the habit of eating mozuku was speculated to be a factor in the absorption of fucoidan.

## 1. Introduction

**Figure 1:**
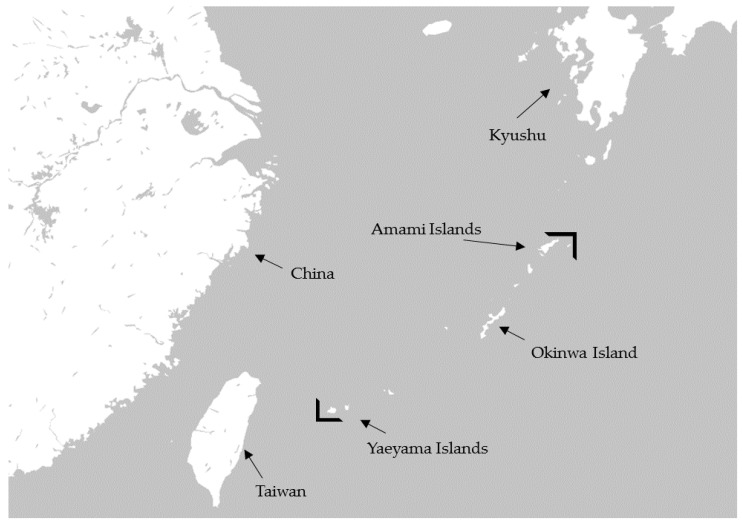
Geographical distribution of Okinawa mozuku.

**Figure 2:**
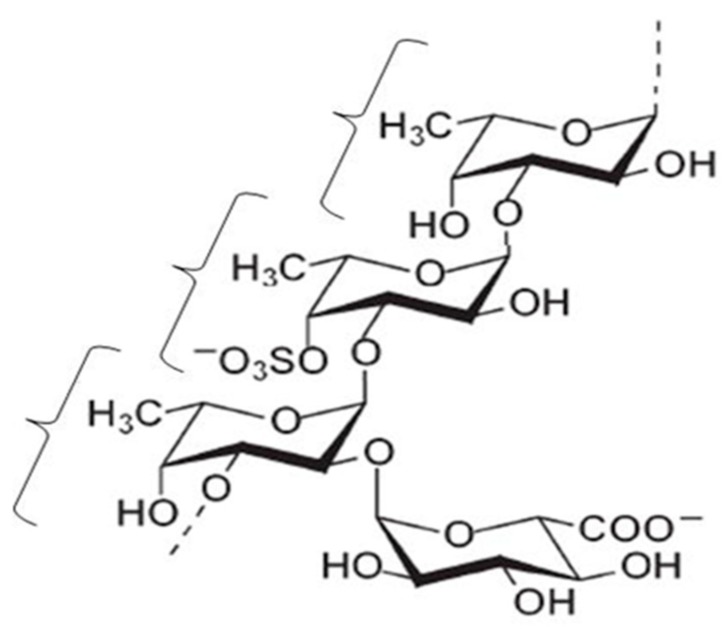
Structure of Okinawa mozuku fucoidan adapted from [4], with permission from Nagaoka, 2012.

According to the National Nutrition Survey in Japan, Japanese people eat about 14.3 g of seaweed per adult person daily [1], which includes konbu (*Laminaria japonica*), wakame (*Undaria pinnatifida*), hijiki (*Hizikia fusiforme*), nori (*Porphyra tenera*), and mozuku (*Nemacystus decipiens*). Mozuku belongs to the brown seaweeds, and several kinds of mozuku such as ishimozuku (*Sphaerotrichia divaricate*), futomozuku (*Tinocladia crassa*), kuromo (*Hydrilla verticillata*), and Okinawa mozuku (*Cladosiphon okamuranus* Tokida) grow wild on the shores of Japan. Okinawa mozuku is a species endemic to the Ryukyu archipelago, which is located between the southern limit (24° N) of the Yaeyama Islands in Okinawa prefecture and the northern limit (29° N) of Amami Island in Kagoshima prefecture (Figure 1) [2]. Okinawa mozuku has been popular as a food among inhabitants of Okinawa prefecture for a long time [3]. Okinawa mozuku contains about 1% fucoidan, and its structure was determined by Nagaoka et al., in 1999 [4]. Mozuku fucoidan is mainly composed of fucose-enriched sulfated polysaccharide with a molecular weight of 28.8 kDa (Figure 2) [4]. Fucodan exhibits many different biological properties, including anti-inflammatory, anticoagulant, antithrombotic, antiadhesive, antiangiogenic, antiviral, antitumor and antioxidant activities [5,6,7,8,9,10]. Currently, the clinical trials of fucoidan have been reported in patients with osteoarthritis and malignant tumors [11,12,13,14] and in the immunocompromised elderly [15]. The biological properties of fucoidan vary depending on the species, molecular weight, structure, and route of intake [9,16,17,18].

Previously, high molecular weight polysaccharides such as fucoidan were not considered to be absorbed orally in humans, because of the lack of a digestive enzyme [19,20]. On the other hand, several studies have reported its biological effects after oral ingestion, but it is unclear how these systemic effects occur. In 2005, Irhimeh et al. [21] reported intestinal absorption of fucoidan using volunteers for the first time. The volunteers took 3 g of Galactofucan (containing 10% and 75% fucoidan) per day for 12 days and showed a 0.6% absorption rate of fucoidan via the intestine. We raised a specific antibody for fucoidan extracted from Okinawa mozuku, and established a sandwich enzyme-linked immuno sorbent assay (ELISA) method for a fucoidan assay [22]. Using this antibody and the ELISA assay, we investigated intestinal absorption of mozuku fucoidan in rats, and showed that ingested fucoidan was absorbed across the intestinal tract and taken up by intestinal macrophages and hepatic Kupffer cells [23]. In addition, we assayed fucoidan concentrations in serum and urine after ingestion of 1 g of mozuku fucoidan using 10 Japanese volunteers. Fucodan was detected in the serum of 7 volunteers and in the urine of 10 volunteers. The rate of absorption through the small intestine was highly variable among the participants. Because it was a preliminary study using a small number of subjects, the mechanism of intestinal absorption of mozuku fucoidan was not elucidated.

In this study, we clarified the factors associated with mozuku fucoidan absorption using larger numbers of Japanese volunteers. Although fucoidan absorption in humans is extremely low, fucoidan concentrations after oral administration were about 10 times higher in urine than in serum. Therefore, urinary fucoidan concentrations were measured before and after an oral administration of mozuku fucoidan.

## 2. Results

### 2.1. Maximum Fucoidan Value in Urine

Urinary fucoidan was detected in 385 out of 396 subjects after a single oral intake of 3 g of fucoidan. Eleven subjects who did not exhibit urinary fucoidan consisted of 3 subjects living in Okinawa prefecture and 8 subjects living outside Okinawa prefecture. Because the maximum value of urinary fucoidan varied widely among subjects, the values were ranked into groups from A to E (Table 1). Fourteen out of 16 subjects in group E (>1200 μg/gCr), which had the highest fucoidan value, lived in Okinawa prefecture. Maximum values of urinary fucoidan (mean ± SD) were as follows: subjects living in Okinawa prefecture (332.3 ± 357.6 μg/gCr) and subjects living outside Okinawa prefecture (240.1 ± 302.4 μg/gCr); males (257.4 ± 310.4 μg/gCr) and females (352.4 ± 373.1 μg/gCr). By age brackets: 309.0 ± 337.4 μg/gCr for subjects in their 20s; 320.0 ± 384.5 μg/gCr for subjects in their 30s; 321.5 ± 392.8 μg/gCr for subjects in their 40s; 249.7 ± 217.6 μg/gCr for subjects in their 50s; 341.3 ± 357.4 μg/gCr for subjects in their 60s; and 153.7 ± 90.0 μg/gCr for subjects in their >70s.

### 2.2. Statistical Analysis

#### 2.2.1. Estimated Urinary Excretion of Fucoidan

Comparing the estimated urinary excretion of fucoidan by place of residence, subjects living in Okinawa prefecture exhibited significantly higher values than those living outside Okinawa prefecture (*p* < 0.01) (Table 2). Gender, age, and habit of eating mozuku were not significant factors associated with the estimated urinary excretion of fucoidan.

#### 2.2.2. Factors Contributing to the Estimated Urinary Excretion of Fucoidan Evaluated by Multiple Regression Analysis

Place of residence of Okinawa prefecture was a significant factor contributing to the estimated urinary excretion of fucoidan (*p* < 0.01), but gender, age, habit of eating mozuku were not significant factors (Table 3). In addition, we evaluated the contributing factors to the estimated urinary excretion of fucoidan in subjects living in Okinawa prefecture and those living outside Okinawa prefecture by multiple regression analysis, respectively. As a result, gender, age, and custom of eating mozuku were not significant factors in both subjects living in Okinawa and living outside Okinawa prefecture (data not shown).

### 2.3. Fucoidan Positive and Negative in Urine before Fucoidan Ingestion

Fucoidan was detected in the urine of 295 subjects (fucoidan positive) but not in 101 subjects (fucoidan negative) before fucoidan ingestion. The backgrounds of both groups are listed in Table 4.

The estimated urinary excretion of fucoidan was significantly higher (*p* < 0.05) in the fucoidan-positive subjects than in the fucoidan-negative subjects. Subjects living in Okinawa prefecture constituted the majority of fucoidan-positive subjects, and the habit of eating mozuku was significantly higher in the fucoidan-positive subjects than the fucoidan-negative subjects.

By multiple regression analysis, the factors relating to the estimated urinary excretion of fucoidan were evaluated in the fucoidan-negative subjects in order to avoid the influence of urinary fucoidan before ingestion on fucoidan absorption. As a result, a subject living in Okinawa prefecture was a significant factor contributing to the estimated urinary excretion of fucoidan (Table 5). However, gender, age, custom of eating mozuku were not significant factors.

## 3. Discussion

The physiological activity of fucoidan differs depending on its molecular weight, structure, and the route of administration. In order to apply fucoidan in a clinical setting, oral intake is more convenient than intravenous injection or intramuscular injection. Fucoidan was absorbed across the intestinal tract in rats and humans [22,23]; however, little is known about the mechanism of its absorption. The present study was conducted to clarify the factors associated with absorption of mozuku fucoidan in 396 Japanese volunteers.

Because 97% of subjects enrolled in this study exhibited urinary fucoidan excretion, intestinal absorption of mozuku fucoidan in humans was reconfirmed. Notably, the estimated urinary excretion of fucoidan was significantly higher in volunteers living in Okinawa prefecture than those living outside Okinawa prefecture (*t*-test). By multiple regression analysis, place of residence of Okinawa prefecture was a significant factor associated with the estimated urinary excretion of fucoidan. As a conceivable reason for this finding, this is a human study on fucoidan absorption using fucoidan derived from Okinawa mozuku. Approximately 90% of Okinawa mozuku is cultivated in Okinawa prefecture, and has been familiar as an edible seaweed among inhabitants of Okinawa prefecture for a long time. The habit of eating mozuku was significantly higher in volunteers living in Okinawa prefecture compared with those living outside Okinawa prefecture, which may reflect the dietary habits of eating mozuku in Okinawa prefecture. The findings suggested the habit of eating mozuku may be associated with fucoidan absorption.

If so, how does the habit of eating mozuku enhance fucoidan absorption? More recently, Tokita et al. reported that Okinawa mozuku was digested and that the fucoidan contained in it was absorbed in humans. The dietary habit of eating mozuku was assumed to be associated with the absorption of fucoidan contained in mozuku [24]. Recently, the transfer of carbohydrate-active enzymes from marine bacteria to Japanese gut microbiota was reported by Hehemann et al. [25]. They showed that digestive enzymes for seaweed were detected frequently in Japanese individuals, whereas those enzymes were absent in North American individuals who did not eat seaweed. In addition, Song T. et al. reported that aga1 (an agarase) may have been transferred together with its surrounding genes, from marine bacteria to soil bacteria via human microbiota. They speculated that microbes from inland humans may degrade agar and that these microbes may have acquired seaweed associated genes because of increased seaweed in diets [26]. Considering these reports, the inhabitants of Okinawa prefecture may have acquired digestive enzymes from mozuku in their gut bacteria because Okinawa mozuku is extensively consumed within this area [3,27].

Of note, urinary fucoidan prior to fucoidan administration was detected in 295 subjects (fucoidan positive) but not in 101 subjects (fucoidan negative). The estimated urinary excretion of fucoidan was significantly higher in the fucoidan positive group than in the fucoidan negative group. In addition, subjects living in Okinawa prefecture and with a habit of eating mozuku were significantly more prevalent in the fucoidan positive group than in the fucoidan negative group. The findings may support the hypothesis that the habit of eating mozuku may be associated with the absorption of fucoidan. The following can be inferred as the reason why urinary fucoidan is detected prior to administration. After oral administration of fucoidan to rats, fucoidan was absorbed and was phagocytosed by hepatic Kupffer cells, and the hepatic fucoidan concentration was increased [24]. Heparin, a polysaccharide similar to fucoidan, was detected in urine 120 h after the oral administration of heparin. Heparin was absorbed through the intestinal tract and taken into the liver immediately, and was then eliminated gradually into the urine [28]. A rapid organ or cellular uptake of fucoidan followed by a slow decrease was noted by Deux et al. [29] although in this case, the fucoidan was delivered intravenously. Similar to the fate after intravenous administration, fucoidan contained in habitually ingested mozuku may be absorbed across the intestinal tract, accumulated in the liver, and then excreted slowly in the urine over a long period of time.

Our study has limitations. First, by multiple regression analysis, the habit of eating mozuku was not a significant factor affecting urinary fucoidan excretion. Second, urinary fucoidan was detected in subjects without the habit of eating mozuku. We speculate the following reason for this. We assayed urinary fucoidan value using polyclonal antibody for Okinawa mozuku fucoidan, which weakly cross-reacted with *Fucus vesiculosus* fucoidan [22]. Since brown seaweeds of konbu (*Laminaria japonica*) and wakame (*Undariapinnatifida*) are traditional foodstuffs in Japan, fucoidan contained in these seaweeds may cross-react with our fucoidan assay method. Further studies are necessary to elucidate the reference of the habit of eating mozuku to intestinal absorption of fucoidan by new ELISA assay using a monoclonal antibody. 

In conclusion, mozuku fucoidan was absorbed after oral ingestion. The habit of eating mozuku was assumed to be associated with the absorption of mozuku fucoidan. The precise mechanism for fucoidan absorption across the intestinal tract may be elucidated in future studies.

## 4. Materials and Methods

### 4.1. Subjects

We published pamphlets describing the purpose, method, exclusion items, etc. of our research entitled ‘The human trial on intestinal absorption of mozuku fucoidan’ on the Internet and recruited volunteer participants (Supplementary Figure S1). Four hundreds and three Japanese people submitted applications from April 2014 to June 2017. They responded to questionnaires on gender, age, residence place, and habit of eating mozuku. We enrolled 396 volunteers who completed questionnaires and collected urine samples as planned.

Age groups were divided into subjects in their 20s, 30s, 40s, 50s, 60s, >70s. Habit of eating mozuku was divided into: (1) almost every day; (2) about 1–3 times a week; (3) about once every 2 weeks; (4) about once a month; (5) about once in 2–3 months; (6) about 1–2 times a year; (7) do not eat/do not like (Table 6).

Residence place of the volunteers was divided into two categories of living in Okinawa prefecture and living outside Okinawa prefecture, because 68% of the participants lived in Okinawa prefecture and the others lived outside Okinawa prefecture. The habit of eating mozuku was significantly higher in volunteers living in Okinawa prefecture than those living outside Okinawa prefecture (Table 6).

This study was carried out in accordance with the Declaration of Helsinki. The protocol of the study was approved by the Ethics Committee of South Product Co., Ltd. (No.14-02). Following an explanation of the study and its aim, all subjects gave informed consent.

Subjects refrained from consuming marine algae or fucoidan supplements on the day before the test and on the day in order to avoid the effects of existing diet. Subjects took orally two fucoidan drinks (1500 mg/bottle) at 9:00 in the morning. Urine samples were collected four times, before, 3, 6, and 9 h after fucoidan administration. Urinary fucoidan concentration was measured using an ELISA method we developed [22]. In addition, we measured urinary creatinine concentration (ELISA method) and corrected urinary fucoidan concentration. In this study, the subjects took orally 3 g of mozuku fucoidan. This dose level was chosen because it was safe and urinary excretion of fucoidan showed dose dependency, which was increased more by administration of 3 g of fucoidan than by 1 or 2 g of fucoidan (Supplementary Figure S2).

Amount of urinary excretion of fucoidan was calculated as follows

[1]. Urinary excretion of fucoidan before fucoidan ingestion.

Urinary fucoidan value before fucoidan ingestion was corrected by urinary creatinine value (μg/gCr), which was matched with urinary excretion of fucoidan for 24 h. Three-eighths of the creatinine correction value was equivalent to the amount of urinary excretion of fucoidan for 9 h.

[2]. Total amount of urinary excretion of fucoidan after fucoidan ingestion.

The creatinine correction value of fucoidan at 3 h after fucoidan ingestion was calculated and one-eighth of it was the equivalent of the amount of urinary excretion of fucoidan for the first 3 h. The creatinine correction value of fucoidan at 6 h after fucoidan ingestion was calculated and one-eighth of it was the equivalent amount to the urinary excretion of fucoidan from 3 to 6 h. Similarly, one-eighth of the creatinine correction value of fucoidan at 9 h was equivalent to the amount of urinary excretion of fucoidan from 6 to 9 h. The total amount of urinary excretion of fucoidan at 3, 6, and 9 h served as the amount of urinary excretion of fucoidan for 9 h after fucoidan ingestion.

[3]. Estimated urinary excretion of fucoidan for 9 h after fucoidan ingestion.

The change in urinary fucoidan excretion before and after fucoidan ingestion [2]−[1] was taken as the estimated urinary excretion of fucoidan for 9 h after fucoidan ingestion.

Calculation formula of fucoidan excretion

$$a\times \frac{9h}{24h}=\left[1\right]$$

$$b\times \frac{3h}{24h}+c\times \frac{3h}{24h}+d\times \frac{3h}{24h}=\left[2\right]$$

$$\left[2\right]-\left[1\right]=\left[3\right]{}^{*}$$

*a*: Urinary fucoidan value before fucoidan ingestion
*b*: The creatinine correction value of fucoidan at 3 h after fucoidan ingestion
*c*: The creatinine correction value of fucoidan at 6 h after fucoidan ingestion
*d*: The creatinine correction value of fucoidan at 9 h after fucoidan ingestion
*h*: hour
[1]: Urinary excretion of fucoidan before fucoidan ingestion
[2]: Total amount of urinary excretion of fucoidan after fucoidan ingestion
[3]: Estimated urinary excretion of fucoidan for 9 h after fucoidan ingestion
* If the amount of urinary excretion of fucoidan before ingestion was higher than the total amount of urinary excretion of fucoidan after fucoidan ingestion, the estimated urinary excretion of fucoidan was judged as 0 (zero).

### 4.2. Statistical Analysis

SAS version 9.4 (Statistical Analysis Software 9.4, SAS Institute Inc., Cary, NC, USA) was used to perform statistical analyses. Differences in gender and habit of eating mozuku between subjects living in Okinawa prefecture and those living outside Okinawa prefecture were carried out by means of χ^2^-test, and age distribution was compared by Wilcoxon rank sum test. Comparison between subjects who produced urinary fucoidan (fucoidan positive) and subjects who did not produce urinary fucoidan (fucoidan negative) prior to taking fucoidan was also carried out by the same statistical analysis.

Correlations between the estimated urinary excretion of fucoidan and gender, age, place of residence, and habit of eating mozuku were analyzed as follows. Gender and place of residence were examined using a two-sample *t*-test; age and habit of eating mozuku were examined using one-way analysis of variance (ANOVA).

In addition, multiple regression analysis was performed with a dependent variable as the estimated urinary excretion of fucoidan, and the independent variable as gender, age, place of residence, and habit of eating mozuku. For all tests, *p* < 0.05 was regarded as significant.

## Figures and Tables

**Table 1:** Maximum value of urinary fucoidan (A: 0–149, B: 150–299, C: 300–699, D: 700–1199, E: >1200 (µg/gCr)).

Distribution of Urinary Fucoidan	A	B	C	D	E
Number (%)	156 (39.4)	112 (28.3)	91 (23.0)	21 (5.3)	16 (4.0)

**Table 2:** Comparison of the estimated urinary excretion of fucoidan.

Parameters	The Estimated Urinary Excretion of Fucoidan (µg/gCr), (Mean ± Standard Deviation (SD))	*p*-Value
In total (n = 396)	57.1 ± 68.9	
Gender: *t*-test		
Male (n = 227)	54.0 ± 69.6	
Female (n = 169)	61.2 ± 68.7	n.s ^1^
Age distribution (years): one-way analysis of variance		
20–29 (n = 81)	53.9 ± 57.0	
30–39 (n = 99)	62.3 ± 74.5	
40–49 (n = 81)	63.9 ± 85.3	
50–59 (n = 74)	47.7 ± 48.4	
60–69 (n = 48)	62.1 ± 78.3	
>70 (n = 13)	30.4 ± 21.3	n.s ^1^
Residence place: *t*-test		
Okinawa prefecture (n = 272)	64.5 ± 72.3	
Outside Okinawa prefecture (n = 124)	40.9 ± 55.9	*p* < 0.01
Habit of eating mozuku: one-way analysis of variance (ANOVA)		
Almost every day (n = 4)	35.9 ± 54.6	
About 1–3 times a week (n = 45)	46.6 ± 39.6	
About once every 2 weeks (n = 69)	57.0 ± 55.0	
About once a month (n = 90)	61.5 ± 72.0	
About once in 2–3 months (n = 88)	69.3 ± 86.9	
About 1–2 times a year (n = 85)	49.2 ± 70.6	
Do not eat/do not like (n = 15)	40.9 ± 40.3	n.s ^1^

^1^ not significant.

**Table 3:** Multiple regression analysis.

Target Variables	*t*-Value	*p*-Value
Residence place	2.69	0.008
Gender	0.77	0.442
Age distribution	−0.76	0.449
Habit of eating mozuku	0.49	0.627

**Table 4:** Comparison between the fucoidan detectors and the fucoidan non-detectors in urine before ingestion.

**Fucoidan Detectors and Non-Detectors**	**Fucidan Positive (µg/gCr)**	**Fucidan Negative (µg/gCr)**	***p*-Value**
Estimated urinary excretion of fucoidan (mean ± SD): *t*-test	64.7 ± 35.7	34.9 ± 35.7	*p* < 0.01
**Parameters**	**Number (%)**	**Number (%)**	***p*-Value**
Gender: χ^2^-test			
Male	168 (56.9)	59 (58.4)	
Female	127 (43.1)	42 (41.6)	n.s ^1^
Age distribution (years): Wilcoxon rank sum test			
20–29	57 (19.3)	24 (23.7)	
30–39	78 (26.4)	21 (20.8)	
40–49	66 (22.4)	15 (14.9)	
50–59	54 (18.3)	20 (19.8)	
60–69	31 (10.5)	17 (16.8)	
>70	9 (3.1)	4 (4.0)	n.s ^1^
Residence place: χ^2^-test			
Okinawa prefecture	222 (75.3)	50 (49.5)	
Outside Okinawa prefecture	73 (24.7)	51(50.5)	*p* < 0.001
Habit of eating mozuku: χ^2^-test			
Almost every day	2 (0.7)	2 (2.0)	
About 1–3 times a week	40 (13.6)	5 (5.0)	
About once every 2 weeks	54 (18.3)	15 (14.9)	
About once a month	64 (21.7)	26 (25.7)	
About once in 2–3 months	70 (23.7)	18 (17.8)	
About 1–2 times a year	55 (18.6)	30 (29.6)	
Do not eat/do not like	10 (3.4)	5 (5.0)	*p* < 0.001

^1^ not significant.

**Table 5:** Multiple regression analysis in fucoidan-positive (n = 295) versus fucoidan-negative subjects (n = 101).

Target Variables	*t*-Value	*p*-Value
Residence place	2.20	0.030
Gender	−0.80	0.425
Age distribution	1.50	0.137
Habit of eating mozuku	−0.63	0.531

**Table 6:** Characteristics of the volunteers living in Okinawa prefecture (n = 272) and those living outside Okinawa prefecture (n = 124).

Parameters	Number (%) ^1^	Number (%) ^2^	Number (%) ^3^	*p*-Value
Gender: χ^2^-test				
Male	227 (57.3)	146 (53.7)	81 (65.3)	
Female	169 (42.7)	126 (46.3)	43 (34.7)	*p* < 0.01
Age distribution (years): Wilcoxon rank sum test				
20–29	81 (20.5)	64 (23.6)	17 (13.7)	
30–39	99 (25.0)	59 (21.7)	40 (32.2)	
40–49	81 (20.5)	55 (20.2)	26 (21.0)	
50–59	74 (18.7)	47 (17.3)	27 (21.8)	
60–69	48 (12.0)	36 (13.2)	12 (9.7)	
>70	13 (3.3)	11 (4.0)	2 (1.6)	n.s ^4^
Habit of eating mozuku: χ^2^-test				
Almost every day	4 (1.0)	2 (0.7)	2 (1.6)	
About 1–3 times a week	45 (11.4)	41 (15.1)	4 (3.2)	
About once every 2 weeks	69 (17.4)	56 (20.6)	13 (10.5)	
About once a month	90 (22.7)	60 (22.1)	30 (24.2)	
About once in 2–3 months	88 (22.2)	60 (22.1)	28 (22.6)	
About 1–2 times a year	85 (21.5)	45 (16.5)	40 (32.3)	
Do not eat/do not like	15 (3.8)	8 (2.9)	7 (5.6)	*p* < 0.01

^1^ total characteristics of the subjects; ^2^ living in Okinawa prefecture; ^3^ living outside Okinawa prefecture; ^4^ not significant.

## Supplementary Materials

The following are available online at http://www.mdpi.com/1660-3397/16/8/254/s1, Figure S1: The study leaflet, Figure S2: Dose dependent study for fucoidan absorption.
