# Supplementary material for: Absorption Study of Mozuku Fucoidan in Japanese Volunteers
Source: Mar Drugs. 2018 Jul 30;16(8):254. doi: 10.3390/md16080254 (PMC6117716; doi:10.3390/md16080254)
Supplement: Supplementary file 1 [file marinedrugs-16-00254-s001.zip › Figure S1.pdf]

Dose dependent study for fucoidan absorption

【Purpose】

This test is conducted to confirm whether the concentration of fucoidan excreted in the urine increases depending on the dose of fucoidan administrated.

【Method】

○ Materials

・Fucoidan drink (one bottle contains 0.5 g fucoidan)

○ Assay

・Sandwich ELISA for fucoidan assay

Subjects took fucoidan drink (fucoidan concentration 1, 2, 3 g). The subjects ingested 1 g of fucoidan at first time. Subjects were allowed to rest for one day, and 2 g of fucoidan was taken at the second time. Subsequently, subjects rested for one day and 3 g of fucoidan was taken for the third time. Urine samples were collected at four times, before, 3, 6, and 9 hours after fucoidan administration. Urinary fucoidan concentration was measured using an ELISA method we developed.

【Results and discussion】

Since the average value of urinary fucoidan concentrations of each intake increased in a dose-dependent manner, it was suggested that fucoidan intake up to 3 g did not reach the plateau (Fig 1).

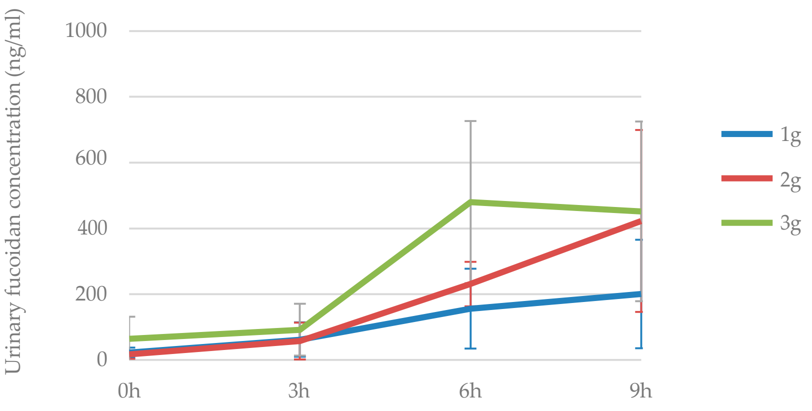

Figure S1. Urinary fucoidan concentration
