# Supplementary material for: Absorption Study of Mozuku Fucoidan in Japanese Volunteers
Source: Mar Drugs. 2018 Jul 30;16(8):254. doi: 10.3390/md16080254 (PMC6117716; doi:10.3390/md16080254)
Supplement: Supplementary file 1 [file marinedrugs-16-00254-s001.zip › Figure S2.pptx]

## Slide 1
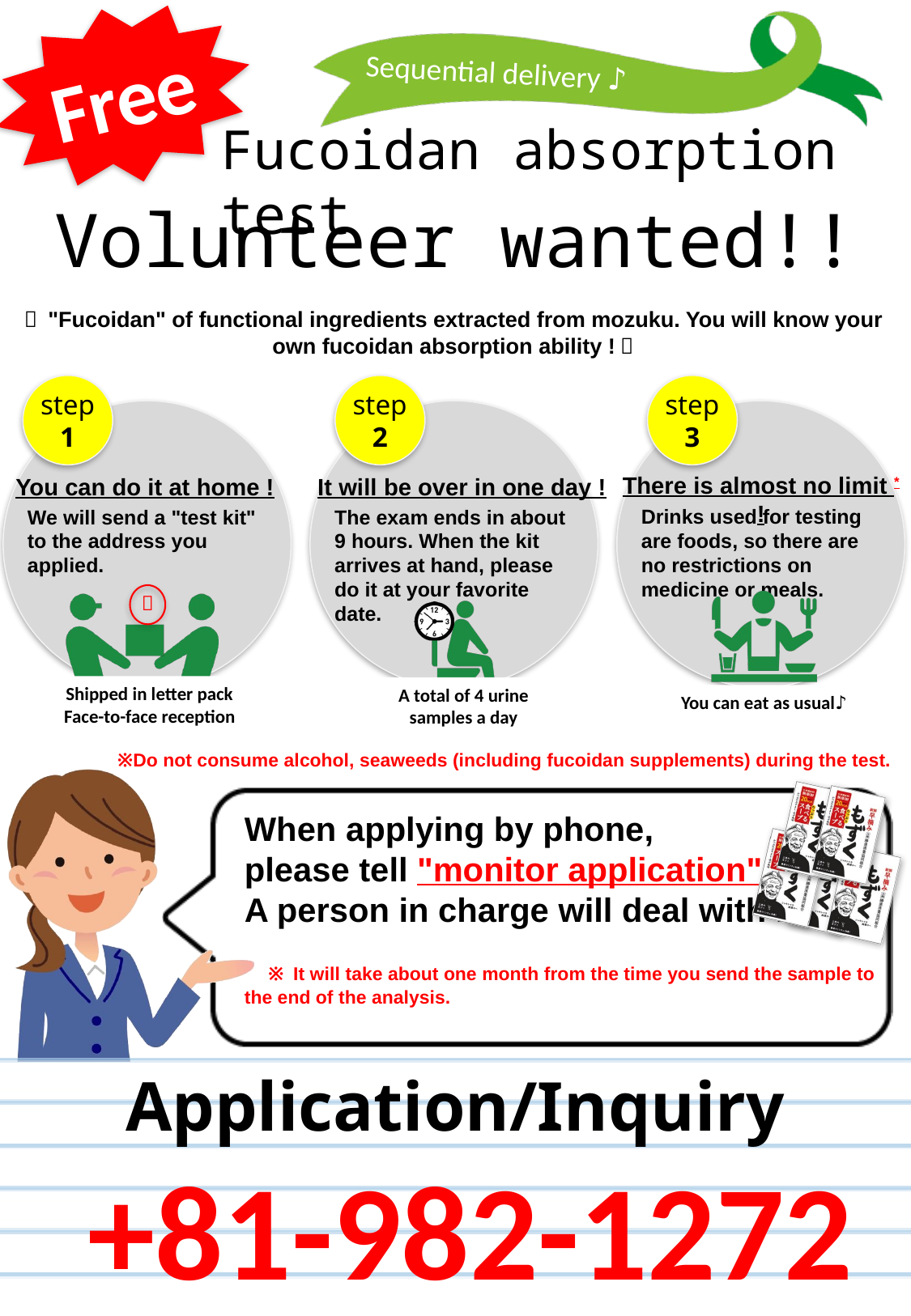

Free
Sequential delivery ♪
Fucoidan absorption test
Volunteer wanted!!
〜 "Fucoidan" of functional ingredients extracted from mozuku. You will know your own fucoidan absorption ability !〜
step
1
step
2
step
3
There is almost no limit * !
You can do it at home !
It will be over in one day !
We will send a "test kit" to the address you applied.
The exam ends in about 9 hours. When the kit arrives at hand, please do it at your favorite date.
Drinks used for testing are foods, so there are no restrictions on medicine or meals.
印
Shipped in letter pack
Face-to-face reception
A total of 4 urine samples a day
You can eat as usual♪
※Do not consume alcohol, seaweeds (including fucoidan supplements) during the test.
When applying by phone,
please tell "monitor application".
A person in charge will deal with it.
　※ It will take about one month from the time you send the sample to the end of the analysis.
Application/Inquiry
 +81-982-1272

## Slide 2
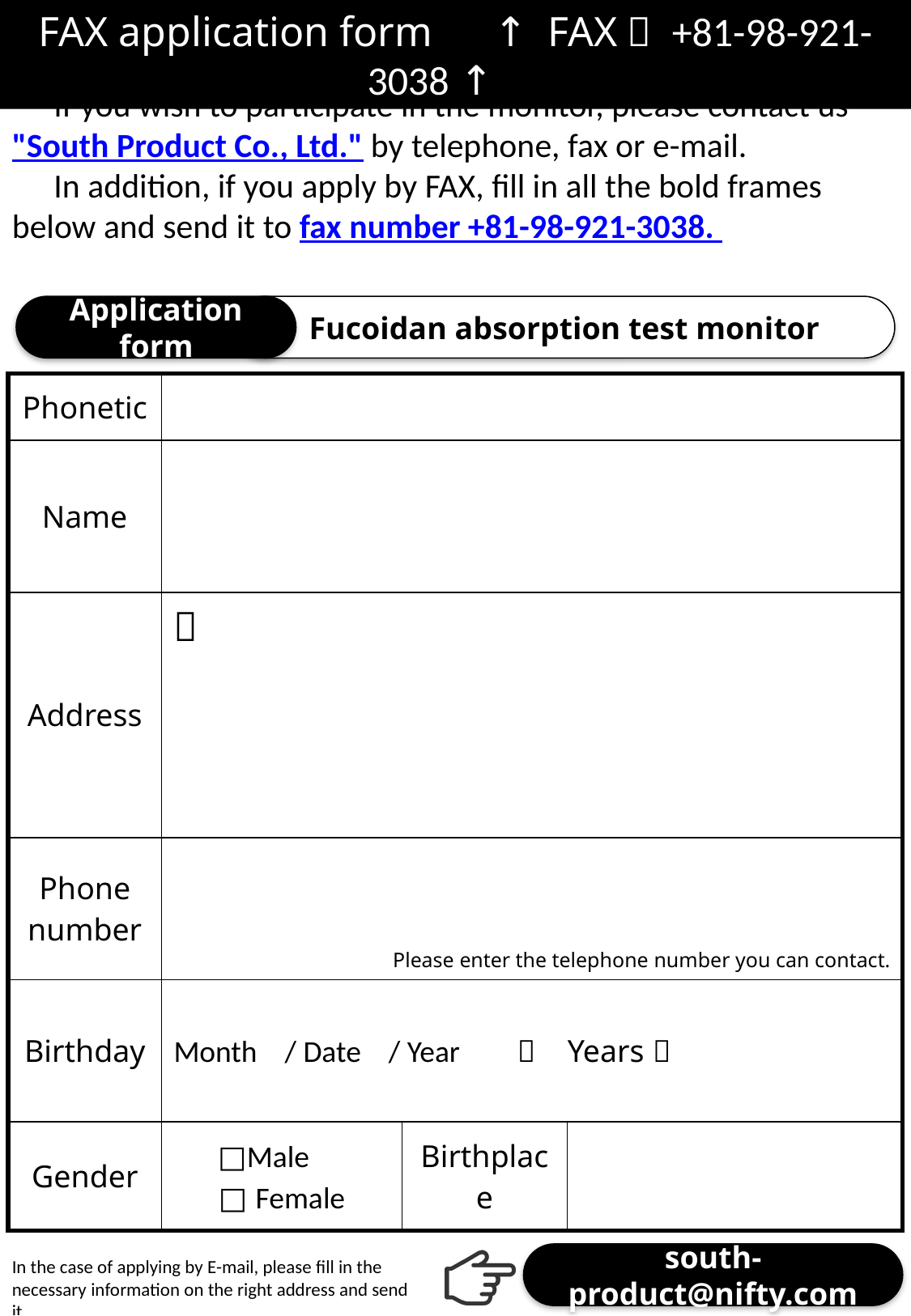

FAX application form 　↑ FAX： +81-98-921-3038 ↑
　If you wish to participate in the monitor, please contact us "South Product Co., Ltd." by telephone, fax or e-mail.
　In addition, if you apply by FAX, fill in all the bold frames below and send it to fax number +81-98-921-3038.
Application form
Fucoidan absorption test monitor
| Phonetic | | | |
| --- | --- | --- | --- |
| Name | | | |
| Address | 〒 | | |
| Phone number | Please enter the telephone number you can contact. | | |
| Birthday | Month / Date / Year 　（ Years） | | |
| Gender | □Male　□Female | Birthplace | |
south-product@nifty.com
In the case of applying by E-mail, please fill in the necessary information on the right address and send it.
